# Effect of Short and Long-Term Treatment with Omega-3 Fatty Acids on Scopolamine-Induced Amnesia

**Published:** 2012

**Authors:** Marjan Ajami, Shariar Eghtesadi, Rouhollah Habibey, Jalaledin mirzay razaz, Habibolah Peyrovi, Mohammadreza Zarrindast, Hamidreza Pazoki-Toroudi

**Affiliations:** a *Department of Nutrition, School of Public health, Tehran University of Medical Sciences, Tehran, Iran.*; b *Physiology Research Center, Tehran University of Medical Sciences, Tehran, Iran. *; c *Department of Community Nutrition, Faculty of Nutrition and Food Technology, Shahid Beheshti University of Medical Sciences and Health Services, Tehran, Iran. *; d *Nano Medicine and Tissue Engineering Center, Shahid Beheshti University of Medical Sciences, Tehran, Iran.*; e *Department of Pharmacology, Tehran University of Medical Sciences, Tehran, Iran.*; f *Institutes for Cognitive Science, Tehran, Iran.*; g *Nano Vichar Pharmaceutical Ltd, Tehran, Iran.*

**Keywords:** Apoptosis, Hippocampus, Memory, Omega-3 fatty acids, Scopolamine

## Abstract

Two omega-3 fatty acids including docosahexaenoic acid (DHA) and eicosapentaenoic acid (EPA) are essential for the physiologic function of neuronal cell membrane. Normal function of neuronal cell membrane requires appropriate composition of fatty in its structure. Present study was designed to compare the effect of short-term and long-term pretreatment with omega-3 fatty acids on scopolamine-induced amnesia and possible involvement of apoptotic or oxidative pathways. Male Wistar rats were gavaged by omega-3 fatty acids [60 mg/Kg (DHA + EPA)] or saline for 2 weeks (short-term model) or 8 weeks (Long-term model), then received intra-CA1 scopolamine (2 µg/rat). Finally, the avoidance response was examined and hippocampus tissue was prepared. Intra-CA1 injection of scopolamine abolished the memory performance in rats. Short-term or long-term pretreatment with omega-3 fatty acids improved memory (p < 0.01 and p < 0.001, respectively). Pretreatment for 2 weeks had no effect on the tissue Malondialdehyde (MDA) contents or SOD and CAT activity. In addition, pretreatment for 2 weeks with omega-3 fatty acids had no effects on tissue Bax and Bcl-2 expression. Conversely, long-term pretreatment with omega-3 fatty acids decreased tissue MDA contents (p < 0.01), SOD activity (p < 0.05) and increased CAT activity (p < 0.01). Long-term pretreatment with omega-3 fatty acids also decreased Bax protein expression (p < 0.05) with no effect on the expression of Bcl-2 protein. In conclusion, long-term exposure to omega-3 fatty acids inhibited the scopolamine-induced oxidative stress, apoptosis and amnesia while the effect of short-term treatment was restricted to the improved memory without significant effect on apoptosis or oxidative stress. Therefore, long-term treatment with low doses of omega-3 fatty acids suggested a suitable treatment for amnesia.

## Introduction

Male Wistar rats weighing 240-300 g were housed at controlled temperature (22°C ± 2°C) on a 12 h alternating light-dark cycle with free access to water and a standard commercial diet with fixed value of carbohydrate, protein, fat, fiber and vitamins plus minerals (660, 230, 40, 60 and 10 mg/Kg, respectively; Nuvital Nutrients, Curitiba, Parana, Brazil). All experimental procedures were conducted in accordance with the NIH Guide for the Care and Use of Laboratory Animals.

*Treatments and surgical procedure*

The drugs used in the present study were scopolamine (Tocris Cookson Ltd., UK), a standardized omega-3 fatty acids formulation, contained 120 mg of DHA and 180 mg of EPA per g (Zahravi Pharmaceutical Company, Tehran, Iran) and for surgical procedures, ketamine and xylazine were used (Alfasan Chemical Co, Woerden, and Holland). Omega-3 fatty acids [60 mg/Kg (DHA: 24 mg/Kg + EPA: 36 mg/Kg)] was administered through gavage in a constant volume of 1 mL for 2 or 8 weeks. Scopolamine (2 µg/rat as 1 µg in each side) was injected bilaterally into the dorsal hippocampal CA1 region in a volume of 1 μL/rat.

Animals were anesthetized with intra-peritoneal injection of ketamine hydrochloride 10% (50 mg/Kg) plus xylazine 2% (4 mg/Kg) and placed in a stereotaxic apparatus. A midline incision was made and the skin and underlying periosteum retracted to expose the rat skull. Bilateral stainless-steel, 22-gauge guide cannulas were placed streotactically 1 mm above the intended site of injection according to the atlas of Paxinos and Watson 2007. Stereotaxic coordinates for the CA1 region of dorsal hippocampus were incisor bar (-3.3 mm), -3 to -3.5 mm (depending on the body weight) posterior to bregma, ±1.8 to 2 mm lateral to the sagittal suture and -2.8 to -3 mm ventral to the dorsal surface of the skull. Cannulas were secured to anchor jewelers’ screws with acrylic dental cement. After completing the surgery, two stainless steel stylets (27-gauge insect pins) were inserted into the guide cannulas to keep them free from debris. After a brief anesthetic clearing period, all animals were allowed to recover from surgery for one week.

The animals were gently restrained by hand; the stylets were removed from the guide cannula and replaced by 27-gauge injection needles (1 mm below the tip of the guide cannula). The injection’s solutions were administered manually in a total volume of 1 µL/rat (0.5 µL in each side) and then animals were immediately placed in their home cages.

*Passive avoidance test*

The step-through inhibitory avoidance apparatus box (Borj Sanat Co, Tehran, Iran) is comprised of two equal compartments, one light (white opaque resin, 20 cm × 20 cm × 30 cm) and one dark (black opaque resin, 20 cm × 20 cm × 30 cm) separated by a guillotine door (7 × 9 cm). Stainless steel grids (2.5 mm in diameter and at 1 cm intervals) were installed on the floor of the dark compartment to produce foot shock. Intermittent electric shocks (50 Hz, 3 s, and 1 mA intensity) were conveyed to the grid floor of the dark compartment by an insulated stimulator.

Training was based on our previous studies (14). Following 30 min of habituation, each animal was gently placed in the light compartment of the apparatus. Five sec later, the guillotine door was opened allowing the animal to enter the dark compartment. The time latency of animal crossing from the light to the dark compartment was recorded and rats with an elapse of more than 100 sec were excluded from the experiments. Once entering the dark compartment, the door was closed and an electrical foot shock (50 Hz, 1 mA and 3 s) was delivered through the stainless steel rods. The training was terminated after the rat remained in the light compartment for a 120 sec period. The number of trials (entries into the dark chamber) was recorded. All the animals were trained with a maximum of 3 trials.

Twenty-four h after the training, a retrieval test was carried out to determine the long-term memory formation. Each animal was placed in the light compartment for 20 sec, then the door was opened and the step-through latency for entering the dark compartment was measured. The cut-off time of 300 sec was applied for those animals which still remained in the light compartment. During these sessions, no electric shock was applied.

Locomotor activity was measured with an activity meter (Borj Sanat Co, Tehran, Iran). On the test day and immediately after the memory retrieval measurement, the animals were placed in the floor of a clear Perspex container (30×25×20 cm high). Horizontal movements were recorded for 5 min by sensors installed beneath the container floor. The number of sensor activations was used as the indicator of animals’ locomotor activity.

Forty-eight rats were randomly divided into 4 groups of 12. Before any pre-training injections, behavioral test and other evaluations, omega-3 fatty acids (60 mg/Kg) were administered through gavage in 2 groups of rats for 14 or 56 days (Table 1). Control groups received diluted solution (saline) without DHA or EPA for 14 or 56 days (Table 1). Scopolamine (2 µg/rat) was injected bilaterally into dorsal hippocampus (CA1 region) in a volume of 1 μL/rat in all groups. Table 1 shows the mentioned designations in detail.

Avoidance response with pre-training administration of scopolamine started immediately after the termination of omega-3 or saline gavage.

**Table 1:** Experimental groups and treatments

**Groups**	**N**	**Weeks of treatment**	**Gavage**	**Pre-training injection**	**Training**	**24 h**	**Behavioral Test**	**Tissue sample**
Saline (2) + Scopolamine	12	2	Saline	Scopolamine	*	-	*	*
Saline (8) + Scopolamine	12	8	Saline	Scopolamine	*	-	*	*
ω3 (2) + Scopolamine	12	2	ω3	Scopolamine	*	-	*	*
ω3 (8) + Scopolamine	12	8	ω3	Scopolamine	*	-	*	*

Gavages: Saline 1 mL or omega-3 fatty acids (ω3) 60 mg/Kg diluted in 1 mL volume. Intra CA1 injections: scopolamine 2 µg/rat (1 µg in each side) or saline (total volume for all treatments was 1 µL).

*Biochemical analysis*

Rats were killed following the passive avoidance test. The brain was rapidly removed and stored at -80°C. Both hippocampuses were carefully separated in physiologic serum. One hippocampus of each rat was homogenized in homogenization buffer (12.5 mM sodium phosphate buffer with pH of 7.0, 400 mM NaCl), followed by centrifugation at 1000 × g for 10 min at 4°C. The resulted supernatant underwent biochemical analysis.

Malondialdehyde (MDA) was measured as an index of lipid peroxidation level in hippocampal tissue. To measure MDA, the method of thiobarbituric acid was used (15), in which MDA reacts with thiobarbituric acid (TBA) to produce a red color with the highest absorbance at 532 nm [Northwest Life Science Specialties LLC (NWLSS^TM^), Malondialdehyde Assay Kit, sensitivity = 0.08 µmol].

Catalase (CAT) and Superoxide Dismutase (SOD) activities were determined using the commercial kits [Catalase Activity Assay Kit (abcam: ab83464) and Superoxide Dismutase Activity Colorimetric Assay Kit (abcam: ab65354)]. CAT activity was measured through a spectrophotometric assay of hydrogen peroxide based on the formation of its yellow stable complex. Catalase first reacted with H_2_O_2_ to produce water and oxygen and the unconverted H_2_O_2_ reacted with OxiRed probe to produce a product measured by colorimetric method at 570 nm. SOD activity was determined by using xanthine oxidase method based on O2•− generation. The rate of the reduction with a superoxide anion is linearly related to the xanthine oxidase (XO) activity and is inhibited with SOD. The inhibition activity of SOD was determined by colorimetric method. The SOD and CAT activities were expressed as units per mg tissue protein (U/mg protein).

*Western blotting for apoptotic or anti-apoptotic protein expression*

The hippocampus from other hemisphere was placed in the solubilization buffer [sodium dodecyl sulfate (2.5%), glycerol (10%), Tris-HCl (62.5 mmol/L, pH = 6.8), and 2-mercaptoethanol (5%)] and boiled for 10 min. The whole extracted tissue was stored at -70°C and the protein concentration was measured using a bicinchoninic acid protein assay (absorbance = 560 nm, Pierce). The equal amount of protein per sample (50 μg) was applied on a 12% sodium dodecyl sulfate polyacrylamide gels with 4.5% stacking gel. After having a rinse in deionized water, the membrane was incubated at 4°C in 0.1 mol/L sodium phosphate buffer (pH = 7.4) and then incubated for 2 h with a 1:3500 dilution of rabbit polyclonal anti-rat Bcl-2 and Bax antibodies [Abcam: Bcl2 antibody and Bax antibody]. Membranes were incubated with HRP-conjugated secondary antibody [Abcam: Rabbit IgG secondary antibody - H and L - Pre-Adsorbed] after three washes. The bands were finally visualized with the ECL chemiluminescence system (Amersham) and the film was developed and used for the measurement of optical density.

**Figure 1:**
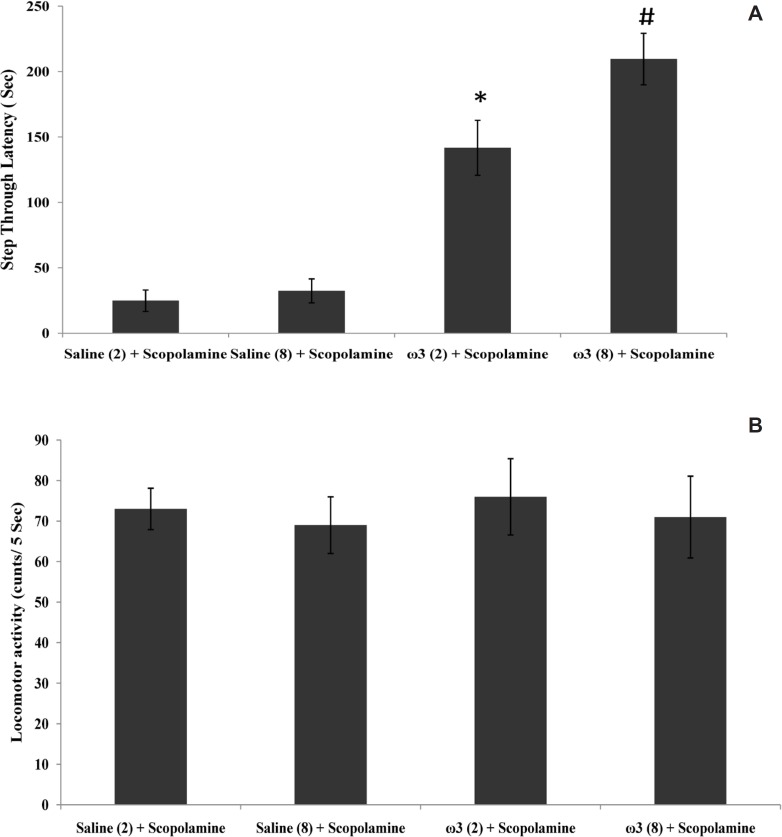
Step trough latency (A) and locomotor activity (B) in scopolamine and omega-3 fatty acids-treated rats. Two weeks (2) or 8 weeks (8) of gavage with saline (1 mL) or omega-3 fatty acids (ω3 60 mg/Kg). Pre-training scopolamine (2 µ/rat) or saline (1 µL/rat) was injected via intra-CA1 root. Data are expressed as mean ± SEM in all groups. *p < 0.01 vs. control group of 2 weeks treatment and #p < 0.001 vs. control group of 8 weeks treatment

*Data analysis*

The results were statistically evaluated by Independent-samples t-test. All data were expressed as mean ± Standard Error of Mean (SEM). In all comparisons, statistical significance level was determind as p < 0.05.

**Figure 2:**
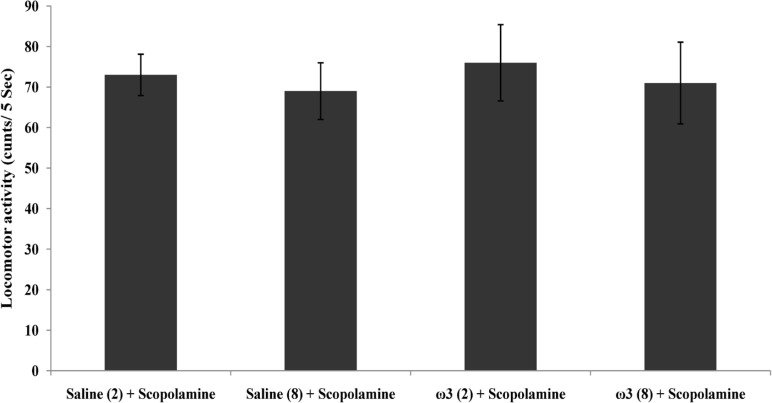
Effect of treatment with scopolamine (2 µ/rat) or omega-3 fatty acids (60 mg/Kg; 2 weeks or 8 weeks) plus scopolamine on the tissue level of Malondialdehyde (MDA). Data are expressed as mean ± SEM in all groups. #p < 0.01 vs. control group of 8 weeks treatment

## Results and Discussion

*The effects of short-term or long-term pre-exposure to omega-3 fatty acids on scopolamine-induced amnesia*

As shown in Figure 1A, the amnesia induced by pre-training scopolamine administration was significantly decreased in rats which had previously received 60 mg/Kg of omega-3 once daily for 2 weeks or 8 weeks (p < 0.01 and p < 0.001, respectively vs. scopolamine-treated control groups). Locomotor activity (p = 0.94) in this experiment was not significantly different in rats which received omega-3 fatty acids and saline-treated groups for 2 or 8 weeks (Figure 1B).

*MDA contents and CAT and SOD activity*

Tissue level of MDA in group of rats which were treated for 2 weeks with 60 mg/Kg omega-3 fatty acids remained unchanged compared to the control group (p > 0.05), however, long-term pretreatment with omega-3 fatty acids at dose of 60 mg/Kg for 8 weeks decreased the tissue MDA contents after the scopolamine injection (p < 0.01 vs. saline (8 weeks) + scopolamine group; Figure 2).

**Figure 3:**
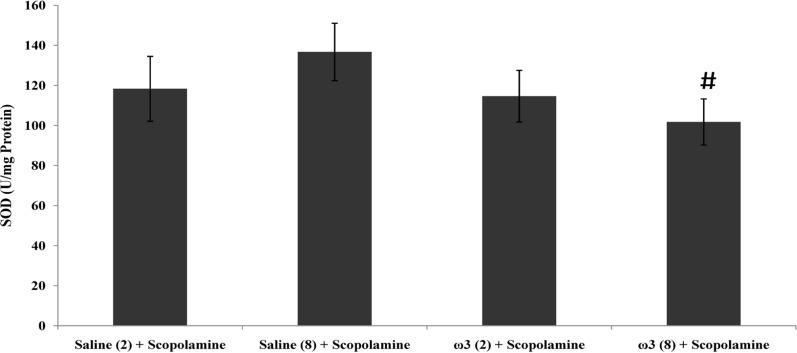
Effect of treatment with scopolamine (2 µg/rat) or omega-3 fatty acids (60 mg/Kg; 2 weeks or 8 weeks) plus scopolamine on the tissue activity of antioxidant enzyme SOD. Data are expressed as mean ± SEM in all groups. #p < 0.05 vs. control group of 8 weeks treatment

SOD activity in hippocampal tissue does not increased through short-term pretreatment with omega-3 fatty acids (60 mg/Kg), while after 8 weeks of treatment, SOD activity was decreased significantly compared with the control group (p < 0.05; Figure 3).

**Figure 4:**
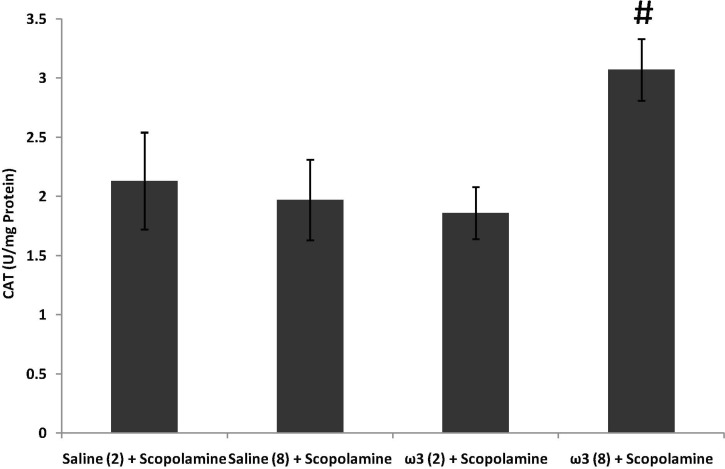
Effect of treatment with scopolamine (2 µg/rat) or omega-3 fatty acids (60 mg/Kg; 2 weeks or 8 weeks) plus scopolamine on the activity of antioxidant enzyme CAT in samples prepared from hippocampus. Data are expressed as mean ± SEM in all groups. #p < 0.01 vs. control group of 8 weeks treatment

CAT activity remained unchanged by short-term pretreatment of omega-3 fatty acids (60 mg/Kg) plus scopolamine (p > 0.05; Figure 4). In long-term omega-3 fatty acids-treated group (60 mg/Kg) CAT activity was significantly increased (p < 0.01 vs. saline (8 weeks) + scopolamine; Figure 4).

*Bax and Bcl-2 expression*

Beta-actin used as a reference protein to evaluate the expression of Bcl-2 or Bax compared to it and presented the value as the ratio of these apoptotic proteins to this protein. The expression of pro-apoptotic protein Bax (21KD) was decreased by the short-term treatment of omega-3 fatty acids (60 mg/Kg), however, it was not statistically significant (p = 0.078; Table 2). Long-term pretreatment with omega-3 fatty acids (60 mg/Kg), significantly decreased the ratio of Bax to beta-actin protein (p < 0.05). The expression of Bcl-2 was not changed significantly using the short or long-term treatment of mega-3 fatty acids at the dose of 60 mg/Kg at present study (Table 2).

**Table 2:** Expression of Bax or Bcl-2 in hippocampus of different groups

	**N**	**Weeks of treatment**	**Bax / ** ***β*** **-actin ratio**	**Bcl-2 / ** ***β*** **-actin ratio**
**Saline (2) + Scopolamine**	12	2	90 ± 11	47 ± 7
**Saline (8) + Scopolamine**	12	8	86 ± 14	51 ± 8
**ω3 (2) + Scopolamine**	12	2	79 ± 15	42 ± 8
**ω3 (8) + Scopolamine**	12	8	61 ± 7 **#**	69 ± 9

Results were expressed as the percentage of *β*-actin expression for each protein in that group (Shown as mean ± SEM). The ratio was obtained by dividing the expression value of each protein to the *β*-actin expression and finally multiplying the result by 100. * p < 0.05 vs. 2 weeks control treatment and # p < 0.05 vs. 8 weeks control treatment.

Dietary omega-3 fatty acids can be hopeful to improve learning and memory (4-6). To our knowledge, for the first time, the present study suggested the beneficial effects of long-term exposure to omega-3 fatty acids on the scopolamine-induced amnesia and its impact on the other molecular events including oxidative stress and apoptosis while the effect of short-term treatment was significant just on the behavioral test.

Reduced latency time for entering the dark part was concomitant with the increased tissue MDA contents and increased SOD activity in hippocampal tissue of rats which were treated with saline and scopolamine. The similar results had been concluded in a very recent study (16) which conducted on the mice brain and confirmed that scopolamine (2 mg/Kg, IP) impairs learning in passive avoidance and the Morris water maze tests, along with the increased MDA contents (lipid peroxidation) and SOD activity but decreased CAT activity. Obvious evidences exist between the oxidative damage of hippocampus and learning impairments (17, 18) by affected gene expression (19). Decreased membrane fluidity is the main consequence of exposure to the reactive oxygen species that impairs cell signaling. In addition, the previous research demonstrated that the overexpression of SOD can impair the LTP formation in CA1 neuronal synapses and consequently affect the memory’s function which is reversible through the treatment of hippocampal slices with catalase (20) or SOD inhibitor (21). The higher levels of CAT activity in the group treated with omega-3 acids for long time was concomitant with the improved memory that is in accordance with the protective effects of CAT on defected memory function.

The previous studies have shown that the overexpression of Bcl-2 not only protects the neural cells against oxidative stress, but also enhances the survival of newborn neurons in primary hippocampal cultures and in adult rat brain (22). The impact of omega-3 fatty acids on the expression of Bcl-2 and Bax proteins has been evaluated in brain (23, 24) which confirms the decreasing of Bax to Bcl-2 ratio in hippocampal tissue after the long-term of pretreatment with omega-3 fatty acids in present study. DHA enhances the expression of anti-apoptotic members of the Bcl-2 gene family and downregulates proapoptotic proteins Bax and Bik (2). In present study, significant changes at molecular level were obtained by long-term treatment with omega-3 fatty acids that emphasized the optimal effect of these agents by their long-term application. It seems that at initial phases, omega-3 fatty acids act by using current cellular resources, while after a long time, it can induce the structural changes which help the synaptic strengthening and learning or memory performance.

In conclusion, we can attribute the protective effects of short-term omega-3 fatty acids treatment on learning and memory to functional factors while after a long time period, the protein translation and the inhibition of neuronal apoptosis and oxidative damage are involved in the anti-amnesic effects of omega-3 fatty acids.
